# The efficacy and safety of *Curcuma longa* extract and curcumin supplements on osteoarthritis: a systematic review and meta-analysis

**DOI:** 10.1042/BSR20210817

**Published:** 2021-06-10

**Authors:** Liuting Zeng, Ganpeng Yu, Wensa Hao, Kailin Yang, Hua Chen

**Affiliations:** 1 Department of Rheumatology and Clinical Immunology, Peking Union Medical College Hospital, Chinese Academy of Medical Sciences and Peking Union Medical College, Key Laboratory of Rheumatology and Clinical Immunology, Ministry of Education, Beijing, China; 2 The Department of Orthopaedics, People's Hospital of Ningxiang City, Ningxiang, Hunan Province, China; 3 Institute of Materia Medica, Chinese Academy of Medical Sciences and Peking Union Medical College, Beijing, China; 4 Graduate School, Capital Medical University, Beijing, China

**Keywords:** Curcuma longa Extract, Curcumin, Meta-analysis, Osteoarthritis, Systematic review

## Abstract

**Objective:** To assess the efficacy and safety of *Curcuma longa* extract and curcumin supplements on osteoarthritis (OA).

**Methods:** The databases such as Pubmed and Cochrane Library were searched to collect the article about *Curcuma longa* extract and curcumin in the treatment of OA. Then, randomized controlled trials (RCTs) were selected and their data were extracted. Finally, the RevMan5.3 was utilized for risk of bias assessment and meta-analysis, the STATA15.0 were utilized for publication bias assessment, and GRADE tool were used for the evidence quality assessment of primary outcomes.

**Results:** A total of 15 RCTs involving 1621 participants were included. (1) Compared with placebo, *Curcuma longa* extract and curcumin (C.) can decrease the visual analog scale (VAS) and The Western Ontario and McMaster Universities (WOMAC) score-pain, the WOMAC score-function and the WOMAC score-stiffness. In terms of adverse events, *Curcuma longa* extract and curcumin are comparable with those of placebo. (2) Compared with non-steroidal anti-inflammatory drugs (NSAIDs), *Curcuma longa* extract and curcumin have similar effects on joint pain, function and stiffness. The incidence of adverse events in *Curcuma longa* extract and curcumin was lower. (3) Compared with the NSAIDs group, C.+NSAIDs can also decrease the VAS and WOMAC score-pain, the WOMAC score-function and the WOMAC score-stiffness. In terms of adverse events, the addition of *Curcuma longa* extract and curcumin to NSAIDs did not increase adverse events.

**Conclusion:** *Curcuma longa* extract and curcumin may be a safer and effective supplement for OA patients. It is recommended to use *Curcuma longa* extract and curcumin supplement for OA patients for more than 12 weeks.

## Introduction

With the increase in the aging population in China, the incidence of osteoarthritis (OA), a chronic degenerative disease, has increased year by year. There are more than 400 million OA patients worldwide, and the disability rate of OA may be as high as 53% [1]. In 2016, the prevalence of symptomatic knee OA in China has reached 8.1%, and the number of patients is at least 110 million with knee OA [2,3]. The main pathological manifestations of OA include the destruction of articular cartilage, the formation of osteophytes, synovitis and joint space narrowing [4]. In addition, symptomatic knee OA can increase the all-cause mortality rate by nearly double, causing a great burden on medical resources and social diseases [5]. The current management of OA is mainly to evaluate the patient’s pain, joint function and the patient’s expected curative effect after the diagnosis of the disease, in order to develop an individualized treatment plan [6]. The main purpose of the treatment is to relieve pain, delay the progress of joint degeneration, improve or restore joint function to improve the patient’s quality of life, including basic treatment, drug treatment and surgical treatment [7–9]. Among them, basic treatment helps patients recognize and change their bad living habits through health education, and encourages patients to exercise appropriately to increase muscle strength and strengthen joint stability, supplemented by physical therapy to change the local metabolic environment of the joints and relieve pain [8]. Drug treatment mostly chooses topical or oral non-steroidal anti-inflammatory drugs (NSAIDs) to relieve pain and improve joint function; oral or intra-articular injection of drugs can also be used to nourish articular cartilage and lubricate the joint cavity [9]. When non-surgical treatment is ineffective, different surgical methods are selected for patients at different disease stages to achieve the purpose of treatment. Among them, artificial joint replacement is an effective and mature method for treating patients with end-stage disease [9]. In the OA stepped and individualized treatment plan, oral drug treatment plays a pivotal role due to its high degree of acceptability and exact curative effect, but the adverse reactions of long-term use of NSAIDs also plague patients and clinical medical staff [10,11].

*Curcuma longa*, a rooted plant in the ginger family, has become the first choice for alternative medicine due to its anti-inflammatory, antioxidant and digestive properties. Its main ingredient, curcumin, is also a natural active oxygen scavenger and active nitrogen provider, and has been proven to be effective in treating pain caused by arthritis and OA [12]. The main mechanism may be related to the protection of IL-1B-induced apoptotic chondrocytes, improvement of early degenerative changes of articular cartilage, inhibition of the production of cytoplasmic phospholipase A2 (cPLA2), cyclooxygenase 2 (COX-2), 5-lipoxygenase (5-LOX) etc. [12,13]. Recent clinical studies have also shown that curcumin can improve many indicators of OA. A recent meta-analysis showed that curcumin can effectively treat patients with OA, improve The Western Ontario and McMaster Universities (WOMAC) score and visual analog scale (VAS) score, and its side effects are not higher than that of ibuprofen, but only five randomized controlled trials (RCTs) were included, which severely limited its applicability of evidence [14]. Another meta-analysis found that curcumin and frankincense formula can relieve symptoms while reducing safety risks. It may be supplementary evidence for the treatment of knee OA, but the quality of the included RCTs is limited, and the number is too small to make it impossible for definite clinical practice recommendations [15]. With the gradual increase in RCTs [16–20] and the accumulation of evidence, there is an urgent need to update the systematic review and meta-analysis. Therefore, this article will conduct a systematic review and meta-analysis on the efficacy and safety of curcumin intervention in OA based on the latest updated evidence.

## Materials and methods

### Literature search strategy

This systematic review and meta-analysis were conducted strictly in accordance with PRISMA guidelines (see supplementary materials). The Chinese databases [China Biology Medicine (CBM), China National Knowledge Infrastructure (CNKI), VIP Database for Chinese Technical Periodicals, Wanfang Database on Academic Institutions in China] were searched, and the search time range was from their establishment to 6 October 2020. The English databases (Web of Science, Embase, PubMed, MEDLINE Complete, ClinicalTrials.gov) were searched in the same way, and the search time range was from their establishment to 6 October 2020. The Cochran Library were also searched (Issue 10 of 12, November 2020). The search strategy of PubMed and Embase is shown in Supplementary Table S1 as an example.

### Selection criteria

#### Participants

Patients diagnosed with OA by recognized criteria. There were no restrictions on gender, age, ethnicity etc.

#### Intervention

The intervention of the experimental group was *Curcuma longa* extracts and curcumin, which could be used alone or in combination with conventional therapies. The control group was a placebo or conventional therapy.

#### Outcomes

The primary outcomes were pain [VAS and WOMAC score-pain], joint function (WOMAC-function), joint stiffness (WOMAC-stiffness), and adverse events. The secondary outcomes were other assessments score of OA [such as the knee injury and osteoarthritis score (KOOS) (including Function in daily living, Function in sport and recreation, Quality of life)] and biochemical indicators (such as oxidative stress indicators and COX-2 levels).

#### Study design

RCTs, with no limitations to publication time, language, quality and publication status.

#### Exclusion criteria

The exclusion criteria were: (1) *Curcuma longa* extracts and curcumin combined with other unconventional therapies; (2) the participant was not human; (3) non-original research literature.

### Literature screening and data extraction

According to the research objects and methods, the literature is initially screened, and then the full text is read, and then screened again according to the above inclusion and exclusion criteria. The data of all RCTs were independently extracted by two reviewers and cross-checked. If there is a disagreement, it will be resolved through discussion by all reviewers. The extracted data include basic information (author, publication time, age of research object etc.), sample size, intervention measures, intervention time, measurement indicators etc.

### Risk of bias assessment

Two reviewer independently used the Cochrane risk of bias assessment tool to evaluate the quality of RCTs, and if there were disagreements, they were discussed with all reviewers [21]. The tool includes the following six aspects: random sequence generation, allocation concealment, blinding, incomplete outcomes, selective reporting and other biases. Each item was recorded as: low risk of bias, high risk of bias, unclear risk of bias.

### Statistical analysis

RevMan 5.3 was used for risk of bias assessment and meta-analysis. *I^2^* is used to test the specificity between RCTs. When there was homogeneity between RCTs (I% < 50%, *P*>0.1), the fixed-effects model is used for meta-analysis. When there was heterogeneity between RCTs (I% > 50%, *P*<0.1), we would first discuss the source of heterogeneity and conduct subgroup analysis. When the heterogeneity was not reduced, the random-effects model was used for meta-analysis [22]. For continuous variables, if the measurement data units were different or the values differed greatly, the standard mean difference (SMD) was used as the effect size indicator, while in other cases, the mean difference (MD) was used as the effect size indicator. For dichotomous variables, the risk ratio (RR) was used as the effect size indicator. The size of the effect was expressed with a 95% confidence interval (95% CI). The publication bias was detected by STATA 15 with Egger’s method (continuous variable) and Harbord methods (dichotomous variable) for primary outcomes. *P*>0.1 is considered to have no publication bias.

## Results

### Results of the search

**Figure 1:**
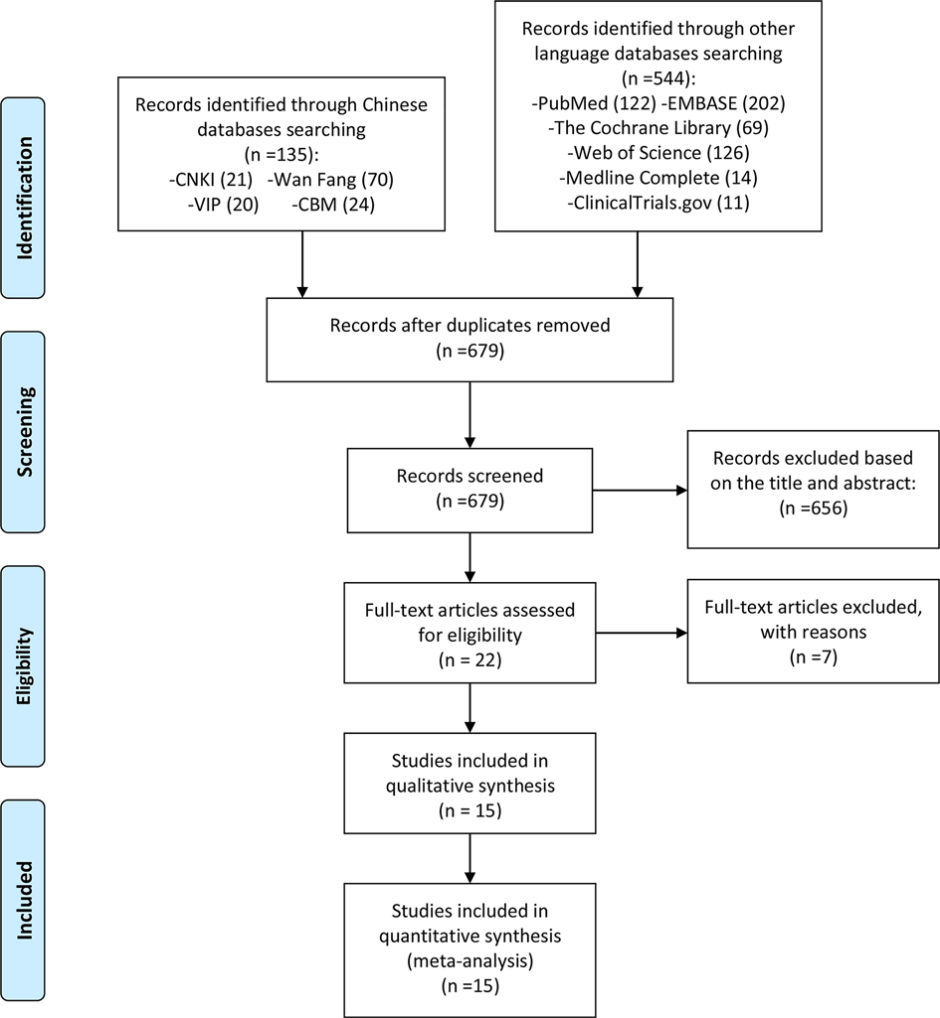
Flow diagram

Of the 679 articles originally included, 22 articles were evaluated in detail, and finally 7 articles were excluded because they did not meet the inclusion and exclusion criteria. In the end, a total of 15 RCTs were included (Figure 1). Among the excluded articles, four were *Curcuma longa* extracts and curcumin combined with other unconventional therapies [23–26], while two of them were not RCTs [27,28], one of them was not original article [29]. The basic characteristics of each RCTs are shown in Table 1.

**Table 1:** The characteristics of the included studies

Study	Country	Sample size (Female/male)		Intervention		
		Trial group	Control group	Trial group	Control group	
Wang et al., 2020 [16]	Australia	36 (18/18)	34 (21/13)	*Curcuma longa* extract 1000 mg	Placebo	
Jamali et al., 2020 [17]	Iran	36 (22/14)	36 (23/13)	Curcumin ointment	Placebo (Vaseline ointment)	
Henrotin et al., 2019 [18]	Belgium	96 (79/17)	45 (34/11)	*Curcuma longa* extract 280 or 197 mg	Placebo	
Hashemzadeh et al., 2020 [19]	Iran	36 (29/7)	35 (31/4)	Curcuminoids (SinaCurcumin™) 40 mg	Placebo	
Shep et al., 2019 [20]	India	70 (48/21)	69 (45/25)	Curcumin (BCM-95®) 1500 mg	Diclofenac sodium 100 mg	
Panahi et al., 2016 [30]	Iran	19 (14/5)	21 (17/4)	Curcuminoids (C3 complex®) 1500 mg	Placebo (inert starch)	
Haroyan et al., 2018 [31]	Armenia	66 (62/5)	68 (65/3)	Curcuminoids 999 mg (CuraMed® 1500 mg)	Placebo	
Kertia et al., 2012 [32]	Indonesia	39 (24/15)	41 (29/12)	Curcuminoid 90 mg	Diclofenac sodium 90 mg	
Kuptniratsaikul et al., 2009 [33]	Thailand	52 (41/11)	55 (45/10)	*Curcuma longa* extract 2000 mg	Ibuprofen 800 mg	
Panahi et al., 2014 [34]	Iran	19 (14/5)	21 (17/4)	Curcuminoid 1500 mg	Placebo (inert starch)	
Kuptniratsaikul et al., 2014 [35]	Thailand	171 (157/14)	160 (139/21)	*Curcuma longa* extract 1500 mg	Ibuprofen 1200 mg	
Nakagawa et al., 2014 [36]	Japan	18 (14/4)	23 (18/5)	Curcumin 180 mg	Placebo	
Pinsornsak et al., 2012 [37]	Thailand	44	44 (total: 62/13)	Curcumin 1000 mg+diclofenac 75 mg	Diclofenac 75 mg	
Madhu et al., 2013 [38]	India	60 (41/19)	60 (42/18)	*Curcuma longa* extract 1000 mg or *Curcuma longa* extract 1000 mg+Glucosamine 1500 mg	Glucosamine 1500 mg or Placebo (Microcrystalline cellulose) 800mg	
Srivastava et al., 2016 [39]	India	78 (53/25)	82 (50/32)	*Curcuma longa* extract 500 mg+Diclofenac 50 mg	Placebo 500 mg+Diclofenac 50 mg	

Study	Relevant outcomes	Mean age (years)		BMI		Duration
		Trial group	Control group	Trial group	Control group	
Wang et al., 2020 [16]	VAS, WOMAC score, adverse events	61.3 ± 8.5	62.4 ± 8.8	29.9 ± 6.3	30.6 ± 7.2	12 weeks
Jamali et al., 2020 [17]	VAS, adverse events	68.86 ± 6.27	67.94 ± 6.72	27.59 ± 3.43	27.54 ± 3.96	6 weeks
Henrotin et al., 2019 [18]	VAS, KOOS, adverse events	60.9 ± 9.78; 61.4 ± 7.49	63.3 ± 7.69	29.4 ± 4.87; 30.4 ± 5.32	29.4 ± 5.2	12 weeks
Hashemzadeh et al., 2020 [19]	WOMAC score, adverse events	54.11 ± 5.80	56.54 ± 5.77	-	-	6 weeks
Shep et al., 2019 [20]	VAS, KOOS, adverse events	53.09 ± 4.17	52.14 ± 3.76	-	-	4 weeks
Panahi et al., 2016 [30]	SOD, GSH, MDA	57.32 ± 8.78	57.57 ± 9.05	28.75 ± 3.17	29.64 ± 4.46	6 weeks
Haroyan et al., 2018 [31]	WOMAC score, adverse events	54.65 ± 8.84	56.04 ± 8.55	28.33 ± 3.6	28.81 ± 3.36	12 weeks
Kertia et al., 2012 [32]	COX-2	64.05 ± 8.83	64.56 ± 8.86	26.28 ± 3.62	26.44 ± 4.79	4 weeks
Kuptniratsaikul et al., 2009 [33]	VAS, adverse events	61.4 ± 8.7	60.0 ± 8.4	26.4 ± 3.7	26.8 ± 4.8	6 weeks
Panahi et al., 2014 [34]	VAS, WOMAC score, adverse events	57.32 ± 8.78	57.57 ± 9.05	28.75 ± 3.17	28.75 ± 3.17	6 weeks
Kuptniratsaikul et al., 2014 [35]	WOMAC score, adverse events	60.3 ± 6.8	60.9 ± 6.9	26.5 ± 3.7	26.6 ± 4.0	4 weeks
Nakagawa et al., 2014 [36]	VAS, adverse events	71.9 ± 5.3	66.1 ± 7.2	25.1 ± 2.7	24.8 ± 2.3	8 weeks
Pinsornsak et al., 2012 [37]	VAS	-	-	-	-	12 weeks
Madhu et al., 2013 [38]	VAS, adverse events	56.63 ± 10.58; 58.17 ± 9.30	56.80 ± 7.99; 56.77 ± 9.98	27.01 ± 4.60; 27.89 ± 5.20	27.80 ± 3.08; 27.97 ± 4.21	6 weeks
Srivastava et al., 2016 [39]	VAS, WOMAC score, adverse events	50.23 ± 8.08	50.27 ± 8.63	28.32 ± 5.06	27.40 ± 5.76	16 weeks

### Description of included trials

Most of the 15 RCTs were from different countries, among which Haroyan et al. (2018) [31] came from Armenia, Wang et al. (2020) [16] came from Australia, Henrotin et al. (2019) [18] came from Belgium, Kertia et al. (2012) [32] came from Indonesia and Nakagawa et al. (2014) [36] came from Japan. In addition, three RCTs came from India [20,38–39], three RCTs came from Thailand [33,35,37] and four RCTs came from Iran [17,19,30,34]. These 15 RCTs involved a total of 1621 participants, and the scale of each RCT was 40–400 participants. There were two experimental groups in Henrotin et al. (2019) (high-dose group and low-dose group), so the control group was divided into two small subgroups accordingly, matching the high-dose group and the low-dose group, respectively (Henrotin et al. (2019a) and Henrotin et al. (2019b)). Madhu et al. (2013) had two experimental groups (*Curcuma longa* extract alone and *Curcuma longa* extract+Glucosamine) and two control groups (Glucosamine and Placebo); for the convenience of comparison, we matched the data of *Curcuma longa* Extract with placebo (Madhu et al. (2013a)), and matched the data of Curcuma longa Extract+Glucosamine with Glucosamine (Madhu et al. (2013b)). The details of study characteristics are presented in Table 1.

### Risk of bias of included studies

**Figure 2:**
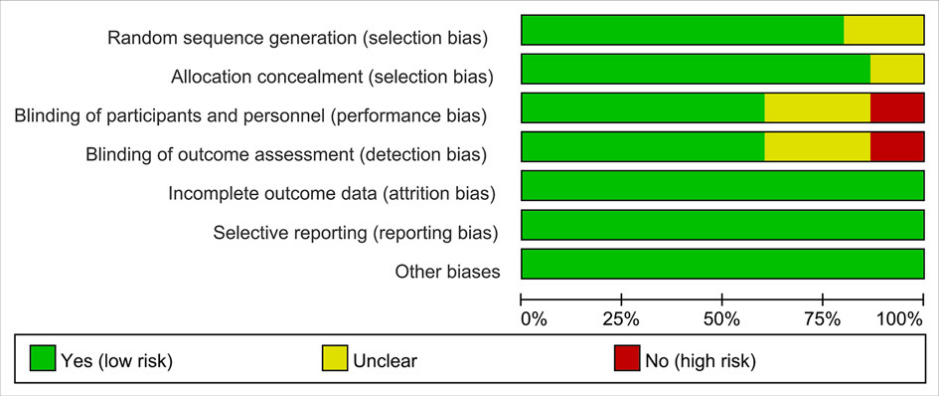
Risk of bias graph

**Figure 3:**
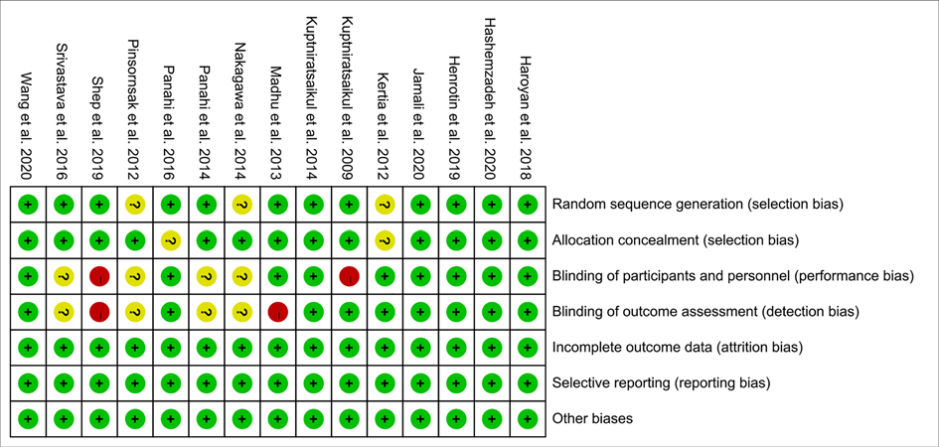
Risk of bias summary

The summary and graph of risk of bias are shown in Figures 2 and 3.

#### Random sequence generation

Three RCTs [32,36,37] did not describe the random sequence generation methods, hence they were rated as unclear risk of bias. Other RCTs described the sequence generation methods, and were rated as low risk of bias. Among those RCTs, Panahi et al. (2016) [30] and Panahi et al. (2014) [34] utilized a 1:1 ratio scheme, Henrotin et al. (2019) [18] utilized blocking randomization, Hashemzadeh et al. (2020) [19] utilized random number table, and Jamali et al. (2020) [17] utilized the online block randomization program. The other seven RCTs used computer software to generate random sequences.

#### Allocation concealment

Two RCTs [30,32] did not describe whether allocation concealment was performed, and therefore were assessed as unclear risk of bias. The remaining RCTs use similar-looking drug packaging, or only allow pharmacists to see the random number, or package the random number in a similar-looking opaque box, or use computer-generated random sequences that cannot be guessed by researchers and participants, so they were considered to be a low risk of bias.

#### Blinding

Kuptniratsaikul et al. (2009) [33] only described the blinding for outcome assessment, but failed to describe the blinding for participants and its outcomes are subjective indicators (VAS), hence it was rated as having low risk of bias in blinding of outcome assessment and having high risk of bias in bling of participants and personnel. Madhu et al. (2013) [38] only used blinding to the participants, not blinding to the measurer, hence it was rated as having low risk of bias in bling of participants and personnel and having high risk of bias in blinding of outcome assessment. Although the four RCTs claimed to use blinding, they did not describe the process of blinding implementation in the paper, so they were assessed as unclear risk of bias. Panahi et al. (2016) [30] and Kertia et al. (2012) [32] did not state whether blinding was used, but because its outcomes are objective indicators (such as COX-2, SOD, MDA), which is less affected by blinding; hence, we assessed the risk of bias as low. Shep et al. (2019) [20] did not mention whether to use blinding, and its main outcome indicators are subjective evaluation indicators, hence it was rated as high risk of bias. Other RCTs describe the process of blind implementation and are therefore judged as low risk of bias.

#### Incomplete outcome data and selective reporting

Although all RCTs exist and participants fall off, because the reasons for falling out and the number of people were balanced, they were considered to be low risk of bias. No selective reports were found in all RCTs, so they were considered low risk of bias.

#### Other potential bias

Other sources of bias were not observed in 15 RCTs; therefore, the risks of other bias of the RCTs were low.

### Primary outcomes

#### Pain

The improvement of pain is represented by the results of VAS and WOMAC score-pain.

**Figure 4:**
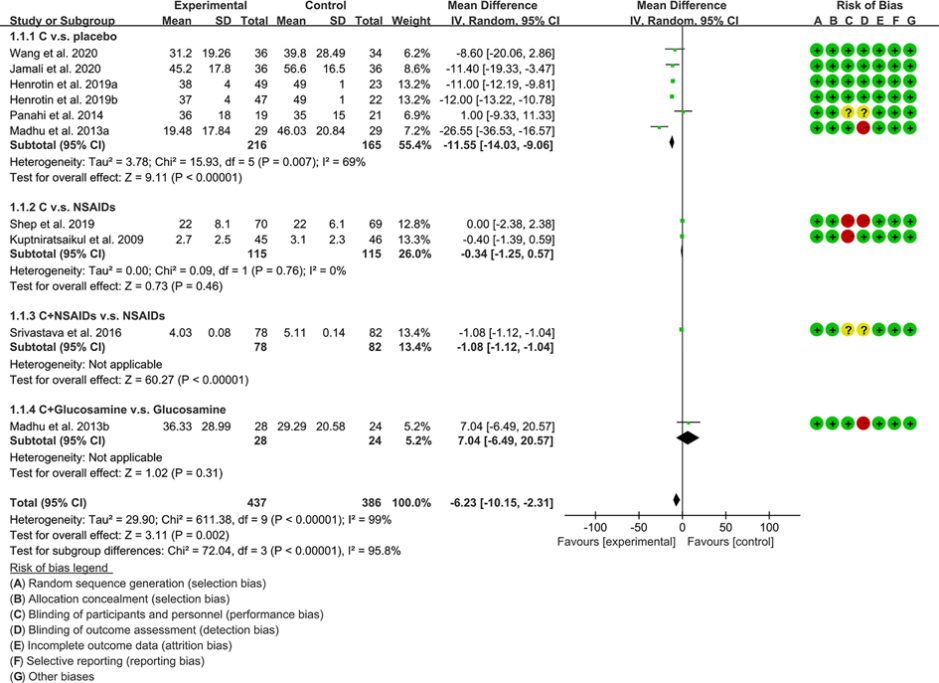
The results of VAS

**Figure 5:**
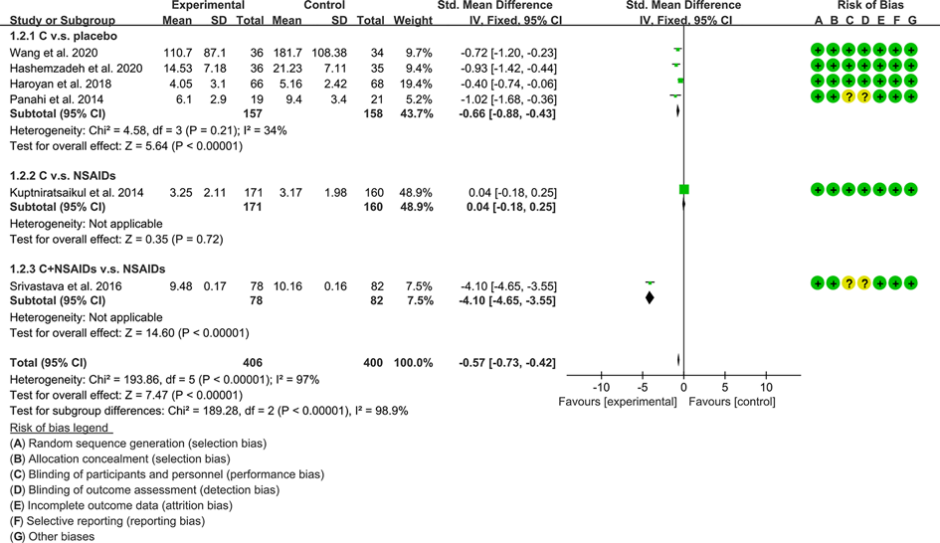
WOMAC score-pain

1. VAS: although ten RCTs reported VAS [16–18,20,33,34,36–39], because the data of Nakagawa et al. (2014) [36] and Pinsornsak et al. (2012) [37] were different from other RCTs, they were not integrated for meta-analysis. These RCTs were divided into different subgroups according to their intervention group and control group: (1) *Curcuma longa* extract and curcumin (C.) v.s. placebo; (2) C. v.s. NSAIDs; (3) C.+ NSAIDs v.s. NSAIDs; (4) C.+Glucosamine v.s. Glucosamine. The heterogeneity test showed that the heterogeneity of the main subgroups was high [(1): *I^2^* = 69%, *P*=0.007; (2): *I^2^* = 0%, *P*=0.76; (3,4): not applicable], so the random-effects model was used for meta-analysis. The meta-analysis results of each subgroup showed that: (1) compared with placebo, *Curcuma longa* extract and curcumin can reduce VAS (WMD: −11.55, 95% CI: −14.3 to −9.06, *P*<0.00001; random-effects model). (2) Compared with the NSAIDs group, there was no statistical difference in the improvement of VAS by *Curcuma longa* extract and curcumin (WMD: −0.34, 95% CI: −1.25 to 0.57, *P*=0.46; random-effects model); (3) Compared with the NSAIDs group, the VAS in C.+NSAIDs group was lower (WMD: −1.08, 95% CI −1.12 to −1.04, *P*<0.00001; random-effects model); (4) The difference of VAS between Glucosamine group and C.+ Glucosamine group was of no statistical significance (WMD: 7.04, 95% CI −6.49 to 20.57, *P*=0.31; random-effects model). The summary result also showed the VAS in experimental group was lower (WMD: −6.23, 95% CI: −10.15 to −2.31, *P*=0.002; random-effects model) (Figure 4).
2. WOMAC score-pain: six RCTs reported WOMAC score-pain of patients [16–18,20,33,34,38,39]. These RCTs are divided into different subgroups according to their intervention group and control group: (1) *Curcuma longa* extract and curcumin (C.) v.s. placebo; (2) C. v.s. NSAIDs; (3) C.+NSAIDs v.s. NSAIDs. The heterogeneity test showed that the heterogeneity of the main subgroups was low [(1): *I^2^* = 34%, *P*=0.21; (2,3): not applicable], so the fixed-effects model was used for meta-analysis. The meta-analysis results of each subgroup showed that: (1) compared with placebo, *Curcuma longa* extract and curcumin can reduce WOMAC score-pain (SMD: −0.66, 95% CI: −0.88 to −0.43, *P*<0.00001; fixed-effects model). (2) Compared with the NSAIDs group, there was no statistical difference in the improvement of WOMAC score-pain by *Curcuma longa* extract and curcumin (SMD: 0.04, 95% CI: −0.18 to 0.25, *P*=0.72; fixed-effects model); (3) compared with the NSAIDs group, the WOMAC score-pain in C.+NSAIDs group was lower (SMD: −4.10, 95% CI −4.65 to −3.55, *P*<0.00001; fixed-effects model). The summary result also showed the WOMAC score-pain in experimental group was lower (SMD: −0.57, 95% CI −0.73 to −0.42, *P*<0.00001; fixed-effects model) (Figure 5).

#### Function

**Figure 6:**
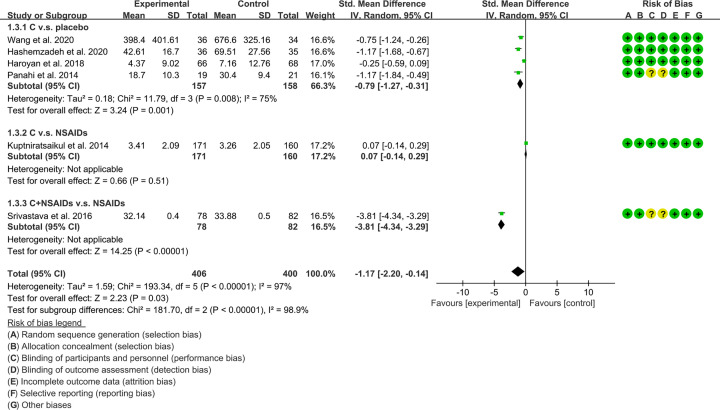
WOMAC score-function

The improvement of function is represented by the results of WOMAC score-function. Six RCTs reported WOMAC score-function of patients [16–18,20,33,34,38,39]. These RCTs are divided into different subgroups according to their intervention group and control group: (1) *Curcuma longa* extract and curcumin (C.) v.s. placebo; (2) C. v.s. NSAIDs; (3) C.+NSAIDs v.s. NSAIDs. The heterogeneity test showed that the heterogeneity of the main subgroups was high [(1): *I^2^* = 75%, *P*=0.008; (2,3): not applicable], so the random-effects model was used for meta-analysis. The meta-analysis results of each subgroup showed that: (1) compared with placebo, *Curcuma longa* extract and curcumin can reduce WOMAC score-function (SMD: −0.79, 95% CI: −1.27 to −0.31, *P*=0.001; random-effects model). (2) Compared with the NSAIDs group, there was no statistical difference in the improvement of WOMAC score-function by *Curcuma longa* extract and curcumin (SMD: 0.07, 95% CI: −0.14 to 0.29, *P*=0.51; random-effects model); (3) compared with the NSAIDs group, the WOMAC score-function in C.+NSAIDs group was lower (SMD: −3.81, 95% CI: −4.34 to −3.29, *P*<0.00001; random-effects model). The summary result also showed the WOMAC score-function in experimental group was lower (SMD: −1.17, 95% CI: −2.20 to −0.14, *P*=0.03; random-effects model) (Figure 6).

#### Stiffness

**Figure 7:**
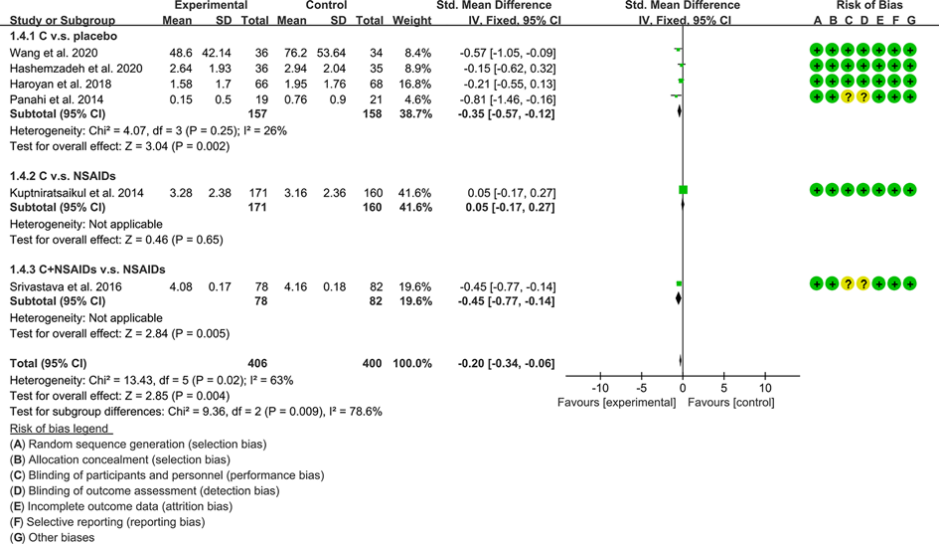
WOMAC score-stiffness

The improvement of function is represented by the results of WOMAC score-stiffness. Six RCTs reported WOMAC score-stiffness of patients [16–18,20,33,34,38,39]. These RCTs are divided into different subgroups according to their intervention group and control group: (1) *Curcuma longa* extract and curcumin (C.) v.s. placebo; (2) C. v.s. NSAIDs; (3) C.+NSAIDs v.s. NSAIDs. The heterogeneity test showed that the heterogeneity of the main subgroups was low [(1): *I^2^* = 25%, *P*=0.25; (2,3): not applicable], so the fixed-effects model was used for meta-analysis. The meta-analysis results of each subgroup showed that: (1) compared with placebo, *Curcuma longa* extract and curcumin can reduce WOMAC score-stiffness (SMD: −0.35, 95% CI: −0.57 to −0.12, *P*=0.002; fixed-effects model). (2) Compared with the NSAIDs group, there was no statistical difference in the improvement of WOMAC score-stiffness by *Curcuma longa* extract and curcumin (SMD: 0.05, 95% CI: −0.17 to 0.27, *P*=0.65; fixed-effects model); (3) compared with the NSAIDs group, the WOMAC score-stiffness in C.+NSAIDs group was lower (SMD: −0.45, 95% CI: −0.77 to −0.14, *P*=0.005; fixed-effects model). The summary result also showed the WOMAC score-stiffness in experimental group was lower (SMD:−0.20, 95% CI: −0.34 to −0.06, *P*=0.004; fixed-effects model) (Figure 7).

### Secondary outcomes

The results of KOOS score and MDA were shown in Table 2. Only Panahi et al. (2016) [30] reported the improvement of SOD and GSH. This RCT found that compared with placebo, the serum SOD activities in curcuminoids group was higher (*P*<0.001). However, the difference of GSH level between curcuminoids group and placebo group was of no statistical significance (*P*=0.064).

**Table 2:** The secondary outcomes

Secondary outcomes	Overall effect						Heterogeneity test			Figure	References
	MD	95% CI	*P*	τ^2^	*I^2^* (%)	*P*	Statistical method	Studies (*n*)	Sample size (*n*)		
KOOS-Function in daily living	−1.67	[−3.27, −0.06]	0.04	-	0	0.94	Fixed effect	2	264	Supplementary Figure S1	[18,20]
KOOS-Function in sport and recreation	−2.48	[−4.26, −0.71]	0.06	-	0	0.49	Fixed effect	2	264	Supplementary Figure S2	[18,20]
KOOS-Quality of life	−1.96	[−7.48, 3.56]	0.49	12.91	52	0.13	Random effect	2	264	Supplementary Figure S3	[18,20]
MDA	−2.06	[−3.80, −0.32]	0.02	1.49	94	<0.0001	Random effect	2	213	Supplementary Figure S4	[30,39]

Only Kertia et al. (2012) [32] reported the improvement of COX-2. This RCT found that the difference in COX-2 between diclofenac sodium group and curcuminoid group was of no statistical significance (*P*=0.89).

### Adverse events

**Figure 8:**
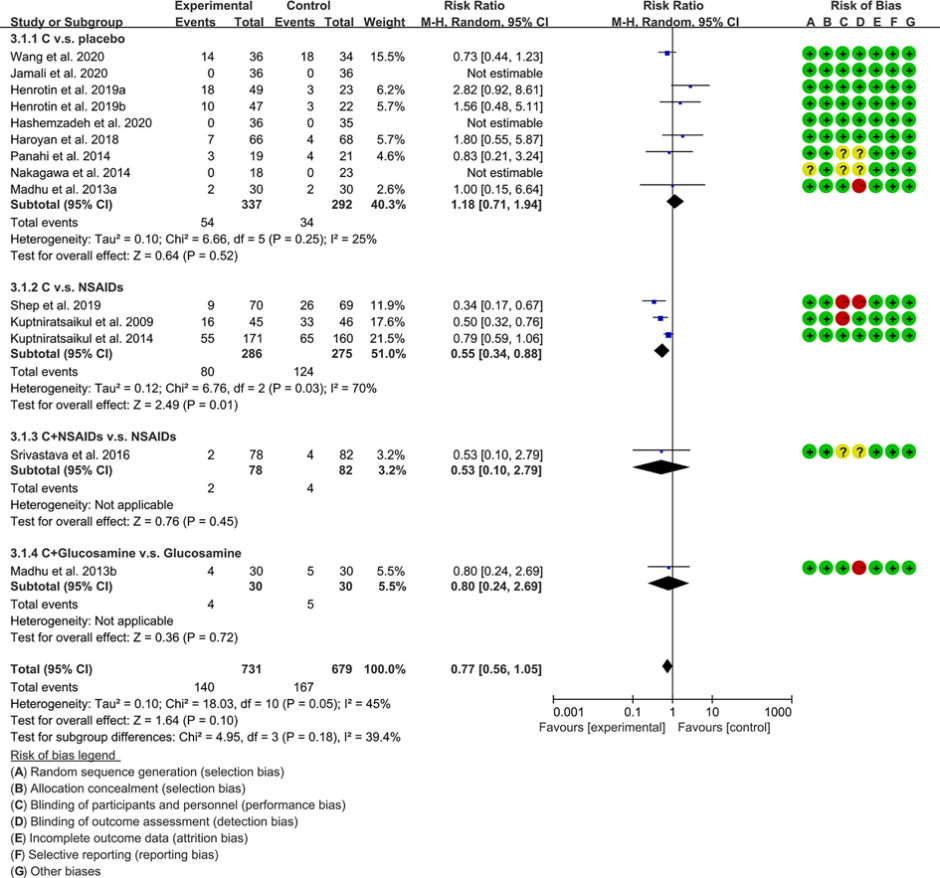
Adverse events

Ten RCTs [16–20,31,33–36,38,39] reported adverse events. These RCTs were divided into different subgroups according to their intervention group and control group: (1) *Curcuma longa* extract and curcumin (C.) v.s. placebo; (2) C. v.s. NSAIDs; (3) C.+NSAIDs v.s. NSAIDs; (4) C.+Glucosamine v.s. Glucosamine. The heterogeneity test showed that the heterogeneity of the subgroups was high [(1): *I^2^* = 25%, *P*=0.25; (2): *I^2^* = 70%, *P*=0.03; (3,4): not applicable], so the random-effects model was used for meta-analysis. The meta-analysis results of each subgroup showed that: (1) the difference of incidence of adverse events between *Curcuma longa* extract and curcumin group and placebo group was of no statistical significance (RR: 1.18, 95% CI: 0.71–1.94, *P*=0.52; random-effects model). (2) Compared with the NSAIDs group, the incidence of adverse events in *Curcuma longa* extract and curcumin was lower (RR: 0.55, 95% CI: 0.34–0.88, *P*=0.01; random-effects model); (3) compared with the NSAIDs group, the incidence of adverse events in C.+NSAIDs group did not increase (RR: 0.53, 95% CI: 0.10–2.79, *P*=0.45; random-effects model); (4) the difference of adverse events between Glucosamine group and C.+Glucosamine group was of no statistical significance (RR: 0.80, 95% CI: 0.24–2.69, *P*=0.72; random-effects model). The summary result also showed the adverse events between in experimental group and control group was of no statistical significance (RR: 0.77, 95% CI: 0.56–1.05, *P*=0.10; random-effects model) (Figure 8).

### Publication bias detection

**Figure 9:**
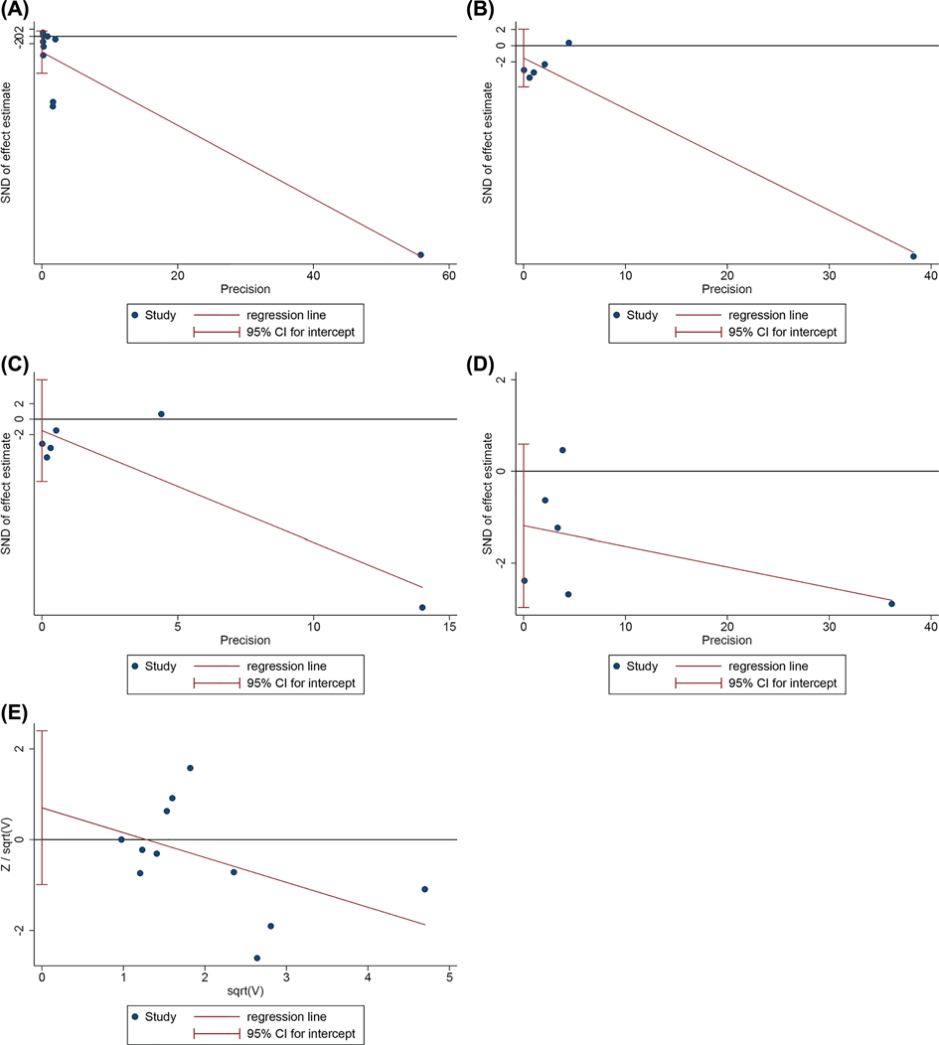
The results of publication bias detection (**A**) VAS; (**B**) WOMAC score-pain; (**C**) WOMAC score-function; (**D**) WOMAC score-stiffness; (**E**) adverse events.

The publication bias of the primary outcomes were detected by STATA 15.0. (1) VAS: The publication bias detection suggests that there may be no publication bias (*P*=0.125) (Figure 9A). (2) WOMAC score-pain: The publication bias detection suggests that there may be no publication bias (*P*=0.301) (Figure 9B). (3) WOMAC score-function: The publication bias detection suggests that there may be no publication bias (*P*=0.565) (Figure 9C). (4) WOMAC score-stiffness: The publication bias detection suggests that there may be no publication bias (*P*=0.138) (Figure 9D). (5) Adverse events: The publication bias detection suggests that there may be no publication bias (*P*=0.372) (Figure 9E).

### Impact of time of treatment

In order to explore the influence of the duration of the intervention on the primary outcomes, we conducted a subgroup analysis of the main results according to the duration of the intervention (Table 3). (1) Pain: VAS showed a difference in the fourth week after the intervention, but there was no difference in the sixth week, and the results after 12 weeks showed a difference again. WOMAC-pain showed different results, there was no difference in the fourth week, and after the sixth week, the results of the two groups showed a difference. (2) WOMAC-function: WOMAC-function showed a difference in the sixth week, but there was no difference between the two groups in the twelfth week. (3) WOMAC-stiffness: the result of WOMAC-stiffness in the sixth week was marginal (*P*=0.05), and the difference began to appear in the twelfth week. (4) Adverse events: there was a difference in the results at the sixth week, but there was no difference in the results at other time points.

**Table 3:** Impact of time of treatment

Duration	Outcomes	Overall effect						Heterogeneity test			Figure
		MD	95% CI	*P*	τ^2^	*I^2^* (%)	*P*	Statistical method	Studies (*n*)	Sample size (*n*)	
4 weeks	VAS	−6.33	[−11.71, −0.96]	0.02	26.43	94	<0.00001	Random	4	352	Supplementary Figure S5
	WOMAC-pain	−0.02	[−0.21, 0.16]	0.82	-	0.29	10	Fixed	2	456	Supplementary Figure S6
	WOMAC-function	0.07	[−0.14, 0.29]	0.51	Not applicable	Not applicable	Not applicable	Random	1	331	Supplementary Figure S7
	WOMAC-stiffness	0.05	[−0.17, 0.27]	0.65	-	Not applicable	Not applicable	Fixed	1	331	Supplementary Figure S8
	Adverse events	0.55	[0.24, 1.26]	0.16	0.29	80	0.02	Random	2	470	Supplementary Figure S9
6 weeks	VAS	−6.26	[−15.91, 3.39]	0.2	99.63	88	<0.00001	Random	4	313	Supplementary Figure S5
	WOMAC-pain	−0.96	[−1.35, −0.57]	<0.00001	-	0	0.83	Fixed	2	111	Supplementary Figure S6
	WOMAC-function	−1.17	[−1.57, −0.76]	<0.00001	0	0	0.99	Random	2	111	Supplementary Figure S7
	WOMAC-stiffness	−0.37	[−0.75, 0.00]	0.05	2.63	62	0.1	Fixed	2	111	Supplementary Figure S8
	Adverse events	0.56	[0.38, 0.82]	0.003	0	0	0.71	Random	5	394	Supplementary Figure S9
8 weeks	Adverse events	Not estimable	Not estimable	Not estimable	Not applicable	Not applicable	Not applicable	Random	1	41	Supplementary Figure S9
12 weeks	VAS	−11.47	[−12.32, −10.62]	<0.00001	0	0	0.46	Random	2	211	Supplementary Figure S5
	WOMAC-pain	−0.5	[−0.78, −0.22]	0.0004	-	10	0.29	Fixed	2	204	Supplementary Figure S6
	WOMAC-function	−0.47	[−0.96, 0.02]	0.06	0.08	63	0.1	Random	2	204	Supplementary Figure S7
	WOMAC-stiffness	−0.33	[−0.61, −0.05]	0.02	-	29	0.24	Fixed	2	204	Supplementary Figure S8
	Adverse events	1.38	[0.68, 2.81]	0.38	0.28	55	0.08	Random	2	345	Supplementary Figure S9
16 Weeks	VAS	−1.08	[−1.12, −1.04]	<0.00001	Not applicable	Not applicable	Not applicable	random	1	160	Supplementary Figure S5
	WOMAC-pain	−4.1	[−4.65, −3.55]	<0.00001	Not applicable	Not applicable	Not applicable	Fixed	1	160	Supplementary Figure S6
	WOMAC-function	−3.81	[−4.34, −3.49]	<0.00001	Not applicable	Not applicable	Not applicable	Random	1	160	Supplementary Figure S7
	WOMAC-stiffness	−0.45	[−0.77, −0.14]	0.005	Not applicable	Not applicable	Not applicable	Fixed	1	160	Supplementary Figure S8
	Adverse events	0.53	[0.10, 2.79]	0.45	Not applicable	Not applicable	Not applicable	Random	1	160	Supplementary Figure S9

## Discussion

For a long time, plant-derived drugs have been highly valued by researchers in the treatment of arthritis. Curcuma, the main active ingredient of *Curcuma longa* extract, is a representative plant-derived medicine. Compared with NSAIDs, it has obvious anti-inflammatory and antioxidant effects and no adverse reactions such as gastrointestinal tract, which indicates that it may become a substitute for NSAIDs [40,41]. A large number of pharmacological studies have also revealed that curcumin has the potential to become a clinical treatment for OA [41–43]. For example, curcumin inhibits inflammation by blocking inflammatory factor-mediated NF-κB, NLRP3 and other signaling pathways, and inhibits oxidation by removing free radicals and enhancing antioxidant enzyme activity, thereby protecting cartilage from damage [44–46]. Curcumin can also promote cartilage matrix repair by adjusting the levels of proteins such as synthin, inhibit chondrocyte apoptosis by promoting autophagy and increasing the activity of anti-apoptotic proteins, and affect chondrocyte proliferation by regulating the Wnt signaling pathway [41–43].

In this systematic review and meta-analysis, we found that: (1) compared with placebo, *Curcuma longa* extract and curcumin can relieve pain (decrease the VAS and WOMAC score-pain), improve the joint function (decrease the WOMAC score-function) and improve the joint stiffness (decrease the WOMAC score-stiffness); in terms of adverse events, *Curcuma longa* extract and curcumin are comparable with those of placebo, suggesting that *Curcuma longa* extract and curcumin are safe. (2) Compared with NSAIDs, *Curcuma longa* extract and curcumin have similar effects on joint pain, function and stiffness. However, the incidence of adverse events in *Curcuma longa* extract and curcumin was lower. (3) Compared with the NSAIDs group, *Curcuma longa* extract and curcumin+NSAIDs can also relieve pain (decrease the VAS and WOMAC score-pain), improve the joint function (decrease the WOMAC score-function) and improve the joint stiffness (decrease the WOMAC score-stiffness); in terms of adverse events, the addition of *Curcuma longa* extract and curcumin to NSAIDs did not increase adverse events; However, due to the small number of RCTs, no definite conclusion can be drawn. (4) The difference of VAS and incident of adverse events between Glucosamine group and C.+Glucosamine group was of no statistical significance. (5) Compared with control group, KOOS-Function in daily living, KOOS-Function in sport and recreation, MDA level in *Curcuma longa* extract and curcumin group is lower. (6) For other oxidative stress indicators (SOD, GSH) and COX-2, since RCTs are less, no definite conclusion can be drawn. (7) In the twelfth week of the intervention, pain, function and stiffness all showed improvement, suggesting that 12 weeks may be an important time point. (8) The heterogeneity of some outcomes are high (such as, adverse events, MDA, VAS etc.). The heterogeneity may be related to the difference in preparation and dosage. According to the subgroup analysis based on the duration of the intervention, although the pain, function and stiffness were inconsistent at the time point before 12 weeks, they all showed improvement after 12 weeks. This suggests that the administration of *Curcuma longa* extract and curcumin must last at least 12 weeks to allow different groups to achieve therapeutic effects. The differences in the results of various indicators at different time points may be related to differences in regions, races, pharmaceutical preparations, drug dosages and so on. Adverse events decreased in the sixth week, and there was no significant difference compared with the control group at other time points. This may indicate that the 6-week-intervention is the time point with the least adverse events, or it may be caused by differences in race, administration methods and pharmaceutical preparations. In the future, it is still necessary to report more outcomes data at different time points of *Curcuma longa* extract and curcumin’s intervention to correct or confirm this result. Current research reports also show that curcumin can inhibit the inflammatory response upstream phospholipase A2 (phospholipase A2, PLA2), COX-2, 5-LOX, iNOS activity. This in turn inhibits the production of inflammatory factors such as midstream IL1β, IL-6, IL-8, TNF-α, and further inhibits the degradation of cartilage matrix by downstream MMP-3, MMP-9 [47–49]. Curcumin can also increase antioxidant enzyme activity and regulate oxidative stress by regulating signal pathways such as Nrf2-ARE, NFκB, MAPK, Notch, AMPK) and NADPH/ROS [50,51].

In addition, we can pay more attention to the role of *Curcuma longa* in OA in the future. *Curcuma longa* contains more phenolic pigments (including curcumin, demethoxycurcumin, bisdemethoxycurcumin) and essential oils (including cineole, linalool, α-terpinene, caryophyllene, ar-curcumene, zingiberen, curcumol, dl-turmerone, arturmerone, dehydrocurdione); it also contains campesterol, stigmasterol, β-sitosterol, cholesterol, fatty acids and metal elements potassium, sodium, magnesium, calcium, manganese, iron, copper, zinc and other multicomponent botanicals [52–55]. Compared with curcumin monomer, because *Curcuma longa* extract contains more components, it may play a multitarget and multisignal pathway transduction role in the treatment of OA pain and inflammation in the molecular pathology mechanism [56,57]. Meanwhile, *Curcuma longa* is a multicomponent botanical drug, and the synergy between its components may bring potential clinical effects in the treatment of OA [58,59]. These components may increase the concentration of each other in the blood of patients with OA through pharmacokinetics and increase the time of each other’s stay in the body, thereby exerting a better clinical effect. Current research showed that the bioavailability of curcumin compound monomers is low [60,61]. However, through the combination with piperine and other substances, the blood concentration of curcumin increased, the elimination half-life was prolonged, the metabolic clearance rate was reduced, and the bioavailability was improved [62–64]. *Curcuma longa* is a multicomponent botanical drug, and the synergy between its different components may also reduce potential side effects. Recent studies have shown that *Curcuma longa* is generally well tolerated even in large doses, although there are still some gastrointestinal side effects, such as nausea and diarrhea, and allergic reactions [65]. Recent studies have also shown the clinical efficacy of *Curcuma longa* extract in OA [16,18]. In the future, the synergistic relationship between the multiple components of *Curcuma longa* can be further explored.

To promote the conclusion, the GRADE tool was utilized to rate the quality of the evidence [66,67]. According to the GRADE handbook [53], the evidence was judged to be high to moderate (Table 4). The quality of WOMAC score-pain and WOMAC score-stiffness was high; the quality of VAS, WOMAC score-function, adverse events was moderate (Table 4).

**Table 4:** Summary of findings for the main comparison

*Curcuma longa* extract and curcumin intervention in patients with OA						
Patient or population: patients with OA						
Outcomes	Illustrative comparative risks^*^ (95% CI)		Relative effect (95% CI)	Number of participants (studies)	Quality of the evidence (GRADE)	Comments
	Assumed risk	Corresponding risk				
	Control	Primary outcomes				
**VAS**		The mean vas in the intervention groups was **6.23 lower** (10.15 to 2.31 lower)		823 (10 studies)	⊕ ⊕ ⊕ ⊖ **moderate**^†^	
**WOMAC score-pain**		The mean womac pain in the intervention groups was **0.57 standard deviations lower** (0.73 to 0.42 lower)		806 (6 studies)	⊕ ⊕ ⊕ ⊕ **high**	SMD −0.57 (−0.73 to −0.42)
**WOMAC score- function**		The mean womac function in the intervention groups was **1.17 standard deviations lower** (2.2 to 0.14 lower)		806 (6 studies)	⊕ ⊕ ⊕ ⊖ **moderate**^†^	SMD −1.17 (−2.2 to −0.14)
**WOMAC score-stiffness**		The mean womac stiffness in the intervention groups was **0.2 standard deviations lower** (0.34 to 0.06 lower)		806 (6 studies)	⊕ ⊕ ⊕ ⊕ **high**	SMD −0.2 (−0.34 to −0.06)
**Adverse events**	**Study population**		RR 0.77 (0.56 to 1.05)	1410 (14 studies)	⊕ ⊕ ⊕ ⊖ **moderate**^†^	
	**246 per 1000**	**189 per 1000** (138 to 258)				
	**Moderate**					
	**133 per 1000**	**102 per 1000** (74 to 140)				

GRADE Working Group grades of evidence.

**High quality:** Further research is very unlikely to change our confidence in the estimate of effect.

**Moderate quality:** Further research is likely to have an important impact on our confidence in the estimate of effect and may change the estimate.

**Low quality:** Further research is very likely to have an important impact on our confidence in the estimate of effect and is likely to change the estimate.

**Very low quality:** We are very uncertain about the estimate.

* The basis for the **assumed risk** (e.g. the median control group risk across studies) is provided in footnotes. The **corresponding risk** (and its 95% CI) is based on the assumed risk in the comparison group and the **relative effect** of the intervention (and its 95% CI).

† Downgraded one level due to the probably substantial heterogeneity.

Some of our results agree with the meta-analysis of Bannuru et al. For example, we have found that *Curcuma longa* extract and curcumin can improve pain, function and stiffness compared with placebo. We also found that there is no difference between *Curcuma longa* extract and NSAIDs in improving pain, function and stiffness. In terms of adverse events, we all found that *Curcuma longa* extract is as safe as placebo and safer than NSAIDs. However, our study included the RCT of *Curcuma longa* extract and curcumin combined with NSAIDs, and showed that this combination is more effective than NSAIDs alone, and the addition of Curcumin does not increase the occurrence of adverse events. Our study also evaluated the effects of *Curcuma longa* extract and curcumin in combination with Glucosamine, and found that the pain improvement and the incidence of adverse events in the *Curcuma longa* extract and curcumin+Glucosamine group were similar to those in the Glucosamine group. However, because there are too few RCTs related to *Curcuma longa* extract and curcumin+Glucosamine and *Curcuma longa* extract and curcumin+NSAIDs, it is not enough to draw a very positive conclusion. In the future, more related RCTs are needed to verify or modify this results. Our meta-analysis also showed that *Curcuma longa* extract and curcumin can improve oxidative stress in patients with OA. Compared with previous meta-analysis, our risk of bias assessment results are different, but we list the reasons for the assessment in detail. And our GRADE score shows that the level of evidence is higher, possibly because our assessment of the risk of bias is lower, and the heterogeneity of RCTs is lower. Our meta-analysis also shows that *Curcuma longa* extract and curcumin may need to be administered for at least 12 weeks to obtain the therapeutic effect. In addition, the RCTs we included are more novel, which increases the reliability of the conclusions. Our meta-analysis shows that the combination of Curcumin and NSAIDs does not increase the occurrence of adverse events and has better efficacy. This is a promising result, because adding Curcumin supplementation in the case of using NSAIDs may increase the efficacy and perhaps reduce the dosage of NSAIDs. This is a direction that can be studied in the future.

In view of the broad prospects of the current development and application of curcumin or *Curcuma longa* extract in the treatment of OA, it is recommended that future RCT research can be in-depth from the following aspects: (1) explore the effects of different administration routes of *Curcuma longa* extract and curcumin (such as oral, topical percutaneous application, joint cavity injection etc.) on its curative effect, and find the best administration method, concentration and dosage of curcumin in the treatment of OA. (2) The role of *Curcuma longa* extract and curcumin combined with other active ingredients (such as quercetin etc.) in the treatment of OA. (3) Report outcomes at different intervention time points. In addition, due to the difference in the incidence of OA between male and female [68], we look forward to future RCTs to analyze the efficacy and safety of different genders, so as to provide more detailed guidance on the medication of patients of different genders.

## Conclusion

This systematic review and meta-analysis show that *Curcuma longa* extract and curcumin can relieve pain and joint stiffness in patients with OA, improve joint function, and would not increase the occurrence of adverse events. Based on current evidence, it is recommended to use *Curcuma longa* extract and curcumin supplement for OA patients for more than 12 weeks. Future RCTs can focus on the different usage and dosage of *Curcuma longa* extract and curcumin, and the curative effect of combination with other drugs.

## Supplementary Material

Supplementary Figures S1-S9 and Table S1

supplementary materials

## Data Availability

The data that support the findings of the present study are openly available in supplementary materials.

## Abbreviations

COX-2: cyclooxygenase 2
GSH: glutathione
IL: interleukin
iNOS: inducible NOS
KOOS: knee injury and osteoarthritis score
MDA: Malondialdehyde
NSAID: non-steroidal anti-inflammatory drug
OA: osteoarthritis
RCT: randomized controlled trial
RR: risk ratio
SMD: standard mean difference
SOD: Superoxide dismutase
VAS: visual analog scale
WOMAC: The Western Ontario and McMaster Universities
5-LOX: 5-lipoxygenase
95% CI: 95% confidence interval
