# Supplementary material for: The efficacy and safety of Curcuma longa extract and curcumin supplements on osteoarthritis: a systematic review and meta-analysis
Source: Biosci Rep. 2021 Jun 10;41(6):BSR20210817. doi: 10.1042/BSR20210817 (PMC8202067; doi:10.1042/BSR20210817)
Supplement: Supplementary Figures S1-S9 and Table S1 [file BSR-2021-0817_supp.pdf]

Study or Subgroup	Experimental Mean	Experimental SD	Experimental Total	Control Mean	Control SD	Control Total	Weight	Mean Difference IV, Fixed, 95% CI	Risk of Bias A	B	C	D	E	F	G
Henrotin et al. 2019a	40.6	20.78	47	41.6	10.85	20	4.4%	-1.00 [-8.61, 6.61]	+	+	+	+	+	+	+
Henrotin et al. 2019b	38.6	23.22	38	41.6	10.85	20	3.3%	-3.00 [-11.78, 5.78]	+	+	+	+	+	+	+
Shep et al. 2019	94.58	6.58	70	96.23	2.71	69	92.2%	-1.65 [-3.32, 0.02]	+	+	−	−	+	+	+
**Total (95% CI)**			**155**			**109**	**100.0%**	**-1.67 [-3.27, -0.06]**							

Heterogeneity: Chi² = 0.12, df = 2 (P = 0.94); I² = 0%

Test for overall effect: Z = 2.04 (P = 0.04)

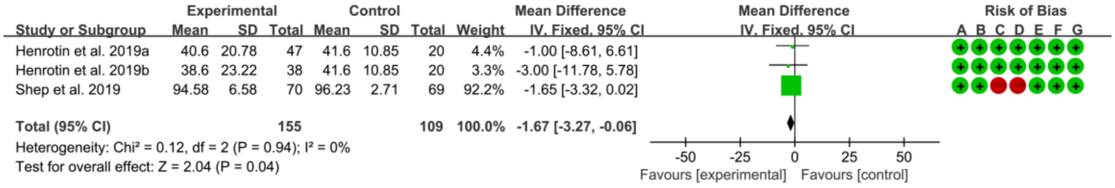

Risk of bias legend

(**A**) Random sequence generation (selection bias)

(**B**) Allocation concealment (selection bias)

(**C**) Blinding of participants and personnel (performance bias)

(**D**) Blinding of outcome assessment (detection bias)

(**E**) Incomplete outcome data (attrition bias)

(**F**) Selective reporting (reporting bias)

(**G**) Other biases

Study or Subgroup	Experimental Mean	Experimental SD	Experimental Total	Control Mean	Control SD	Control Total	Weight	Mean Difference IV, Fixed, 95% CI	A	B	C	D	E	F	G
Henrotin et al. 2019a	8.4	22.73	47	7.5	19.87	20	2.7%	0.90 [-9.97, 11.77]	+	+	+	+	+	+	+
Henrotin et al. 2019b	10.2	17.64	38	7.5	19.87	20	2.9%	2.70 [-7.66, 13.06]	+	+	+	+	+	+	+
Shep et al. 2019	89.14	6.43	70	91.88	4.38	69	94.4%	-2.74 [-4.57, -0.91]	+	+	-	-	+	+	+
**Total (95% CI)**			**155**			**109**	**100.0%**	**-2.48 [-4.26, -0.71]**							

Heterogeneity: Chi² = 1.41, df = 2 (P = 0.49); I² = 0%
Test for overall effect: Z = 2.74 (P = 0.006)

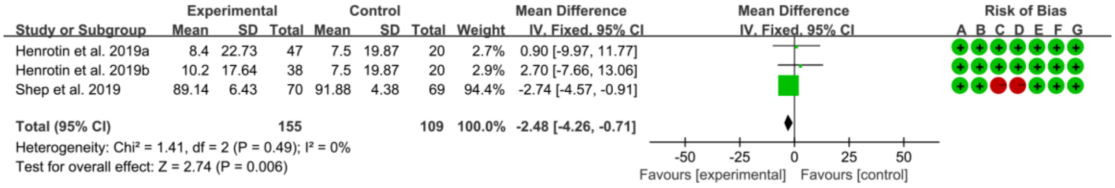

Risk of bias legend
(**A**) Random sequence generation (selection bias)
(**B**) Allocation concealment (selection bias)
(**C**) Blinding of participants and personnel (performance bias)
(**D**) Blinding of outcome assessment (detection bias)
(**E**) Incomplete outcome data (attrition bias)
(**F**) Selective reporting (reporting bias)
(**G**) Other biases

	Experimental			Control				Mean Difference	Risk of Bias						
Study or Subgroup	Mean	SD	Total	Mean	SD	Total	Weight	IV, Random, 95% CI	A	B	C	D	E	F	G
Henrotin et al. 2019a	19.6	21.59	47	26.7	15.09	20	23.2%	-7.10 [-16.15, 1.95]	+	+	+	+	+	+	+
Henrotin et al. 2019b	22.3	19.4	38	26.7	15.09	20	23.2%	-4.40 [-13.44, 4.64]	+	+	+	+	+	+	+
Shep et al. 2019	73.57	7.46	70	72.26	8.61	69	53.6%	1.31 [-1.37, 3.99]	+	+	−	−	+	+	+
**Total (95% CI)**			**155**			**109**	**100.0%**	**-1.96 [-7.48, 3.56]**							

Heterogeneity: Tau² = 12.91; Chi² = 4.15, df = 2 (P = 0.13); I² = 52%
Test for overall effect: Z = 0.70 (P = 0.49)

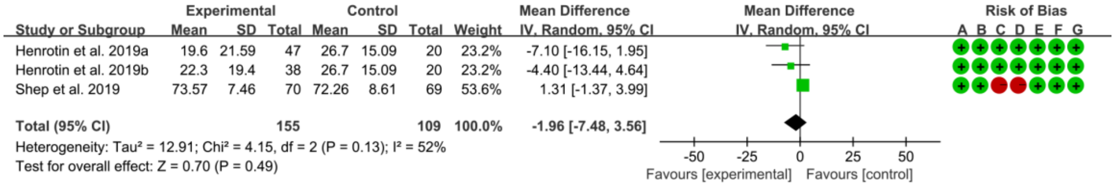

Risk of bias legend
(**A**) Random sequence generation (selection bias)
(**B**) Allocation concealment (selection bias)
(**C**) Blinding of participants and personnel (performance bias)
(**D**) Blinding of outcome assessment (detection bias)
(**E**) Incomplete outcome data (attrition bias)
(**F**) Selective reporting (reporting bias)
(**G**) Other biases

Study or Subgroup	Experimental Mean	Experimental SD	Experimental Total	Control Mean	Control SD	Control Total	Weight	Mean Difference IV, Random, 95% CI	A	B	C	D	E	F	G
Panahi et al. 2016	17	2	27	20	1	26	47.1%	-3.00 [-3.85, -2.15]	+	?	+	+	+	+	+
Srivastava et al. 2016	3.69	0.12	78	4.91	0.11	82	52.9%	-1.22 [-1.26, -1.18]	+	+	?	?	+	+	+
**Total (95% CI)**			**105**			**108**	**100.0%**	**-2.06 [-3.80, -0.32]**							

Heterogeneity: Tau² = 1.49; Chi² = 16.95, df = 1 (P < 0.0001); I² = 94%
Test for overall effect: Z = 2.32 (P = 0.02)

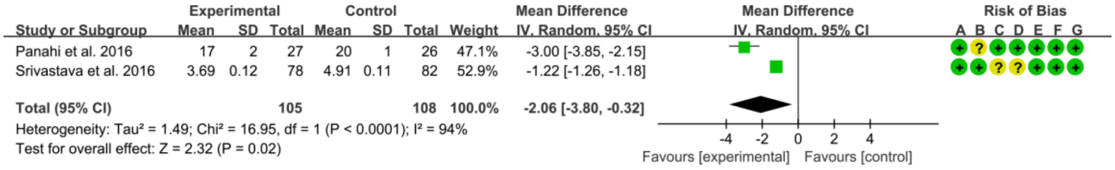

Risk of bias legend

(**A**) Random sequence generation (selection bias)
(**B**) Allocation concealment (selection bias)
(**C**) Blinding of participants and personnel (performance bias)
(**D**) Blinding of outcome assessment (detection bias)
(**E**) Incomplete outcome data (attrition bias)
(**F**) Selective reporting (reporting bias)
(**G**) Other biases

Study or Subgroup	Experimental Mean	Experimental SD	Experimental Total	Control Mean	Control SD	Control Total	Weight	Mean Difference IV, Random, 95% CI
**4.1.1 4 Weeks**								
Shep et al. 2019	22	8.1	70	22	6.1	69	9.4%	0.00 [-2.38, 2.38]
Henrotin et al. 2019a	44	4	49	54	4	23	9.5%	-10.00 [-11.98, -8.02]
Henrotin et al. 2019b	44	4	47	54	4	22	9.5%	-10.00 [-12.03, -7.97]
Jamali et al. 2020	52.2	17	36	56.9	15.2	36	6.7%	-4.70 [-12.15, 2.75]
**Subtotal (95% CI)**			**202**			**150**	**35.2%**	**-6.33 [-11.71, -0.96]**
Heterogeneity: Tau² = 26.43; Chi² = 50.52, df = 3 (P < 0.00001); I² = 94%								
Test for overall effect: Z = 2.31 (P = 0.02)								
**4.1.2 6 Weeks**								
Jamali et al. 2020	45.2	17.8	36	56.6	16.5	36	6.5%	-11.40 [-19.33, -3.47]
Kuptniratsaikul et al. 2009	2.7	2.5	45	3.1	2.3	46	9.8%	-0.40 [-1.39, 0.59]
Panahi et al. 2014	36	18	19	35	15	21	5.2%	1.00 [-9.33, 11.33]
Madhu et al. 2013a	19.48	17.84	29	46.03	20.84	29	5.4%	-26.55 [-36.53, -16.57]
Madhu et al. 2013b	36.33	28.99	28	29.29	20.58	24	3.9%	7.04 [-6.49, 20.57]
**Subtotal (95% CI)**			**157**			**156**	**30.7%**	**-6.26 [-15.91, 3.39]**
Heterogeneity: Tau² = 99.63; Chi² = 34.45, df = 4 (P < 0.00001); I² = 88%								
Test for overall effect: Z = 1.27 (P = 0.20)								
**4.1.3 12 Weeks**								
Wang et al. 2020	31.2	19.26	36	39.8	28.49	34	4.7%	-8.60 [-20.06, 2.86]
Henrotin et al. 2019a	38	4	49	49	1	23	9.7%	-11.00 [-12.19, -9.81]
Henrotin et al. 2019b	37	4	47	49	1	22	9.7%	-12.00 [-13.22, -10.78]
**Subtotal (95% CI)**			**132**			**79**	**24.2%**	**-11.47 [-12.32, -10.62]**
Heterogeneity: Tau² = 0.00; Chi² = 1.57, df = 2 (P = 0.46); I² = 0%								
Test for overall effect: Z = 26.47 (P < 0.00001)								
**4.1.4 16 Weeks**								
Srivastava et al. 2016	4.03	0.08	78	5.11	0.14	82	9.9%	-1.08 [-1.12, -1.04]
**Subtotal (95% CI)**			**78**			**82**	**9.9%**	**-1.08 [-1.12, -1.04]**
Heterogeneity: Not applicable								
Test for overall effect: Z = 60.27 (P < 0.00001)								
**Total (95% CI)**			**569**			**467**	**100.0%**	**-6.85 [-10.29, -3.42]**
Heterogeneity: Tau² = 31.20; Chi² = 763.97, df = 12 (P < 0.00001); I² = 98%								
Test for overall effect: Z = 3.91 (P < 0.0001)								
Test for subgroup differences: Chi² = 578.84, df = 3 (P < 0.00001), I² = 99.5%								

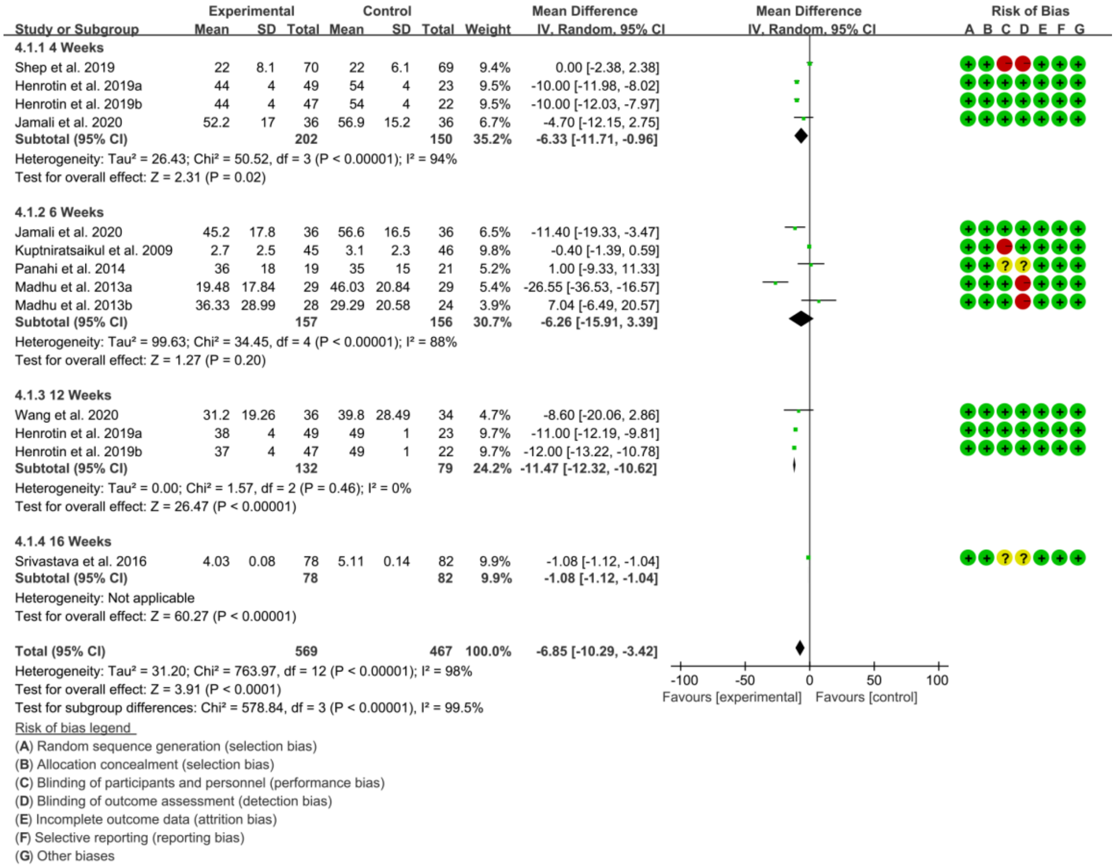

Risk of bias legend
(**A**) Random sequence generation (selection bias)
(**B**) Allocation concealment (selection bias)
(**C**) Blinding of participants and personnel (performance bias)
(**D**) Blinding of outcome assessment (detection bias)
(**E**) Incomplete outcome data (attrition bias)
(**F**) Selective reporting (reporting bias)
(**G**) Other biases

Study or Subgroup	Experimental Mean	Experimental SD	Experimental Total	Control Mean	Control SD	Control Total	Weight	Std. Mean Difference IV, Fixed, 95% CI	Risk of Bias A	B	C	D	E	F	G
**4.2.1 4 Weeks**															
Haroyan et al. 2018	4.37	2.88	60	4.92	3.09	65	15.5%	-0.18 [-0.53, 0.17]	+	+	+	+	+	+	+
Kuptniratsaikul et al. 2014	3.25	2.11	171	3.17	1.98	160	41.3%	0.04 [-0.18, 0.25]	+	+	+	+	+	+	+
**Subtotal (95% CI)**			**231**			**225**	**56.8%**	**-0.02 [-0.21, 0.16]**							
Heterogeneity: Chi² = 1.11, df = 1 (P = 0.29); I² = 10%															
Test for overall effect: Z = 0.23 (P = 0.82)															
**4.2.2 6 Weeks**															
Hashemzadeh et al. 2020	14.53	7.18	36	21.23	7.11	35	8.0%	-0.93 [-1.42, -0.44]	+	+	+	+	+	+	+
Panahi et al. 2014	6.1	2.9	19	9.4	3.4	21	4.4%	-1.02 [-1.68, -0.36]	+	+	?	?	+	+	+
**Subtotal (95% CI)**			**55**			**56**	**12.3%**	**-0.96 [-1.35, -0.57]**							
Heterogeneity: Chi² = 0.05, df = 1 (P = 0.83); I² = 0%															
Test for overall effect: Z = 4.77 (P < 0.00001)															
**4.2.3 12 Weeks**															
Wang et al. 2020	110.7	87.1	36	181.7	108.38	34	8.2%	-0.72 [-1.20, -0.23]	+	+	+	+	+	+	+
Haroyan et al. 2018	4.05	3.1	66	5.16	2.42	68	16.4%	-0.40 [-0.74, -0.06]	+	+	+	+	+	+	+
**Subtotal (95% CI)**			**102**			**102**	**24.6%**	**-0.50 [-0.78, -0.22]**							
Heterogeneity: Chi² = 1.11, df = 1 (P = 0.29); I² = 10%															
Test for overall effect: Z = 3.53 (P = 0.0004)															
**4.2.4 16 Weeks**															
Srivastava et al. 2016	9.48	0.17	78	10.16	0.16	82	6.3%	-4.10 [-4.65, -3.55]	+	+	?	?	+	+	+
**Subtotal (95% CI)**			**78**			**82**	**6.3%**	**-4.10 [-4.65, -3.55]**							
Heterogeneity: Not applicable															
Test for overall effect: Z = 14.60 (P < 0.00001)															
**Total (95% CI)**			**466**			**465**	**100.0%**	**-0.51 [-0.65, -0.38]**							
Heterogeneity: Chi² = 197.89, df = 6 (P < 0.00001); I² = 97%															
Test for overall effect: Z = 7.27 (P < 0.00001)															
Test for subgroup differences: Chi² = 195.62, df = 3 (P < 0.00001), I² = 98.5%															

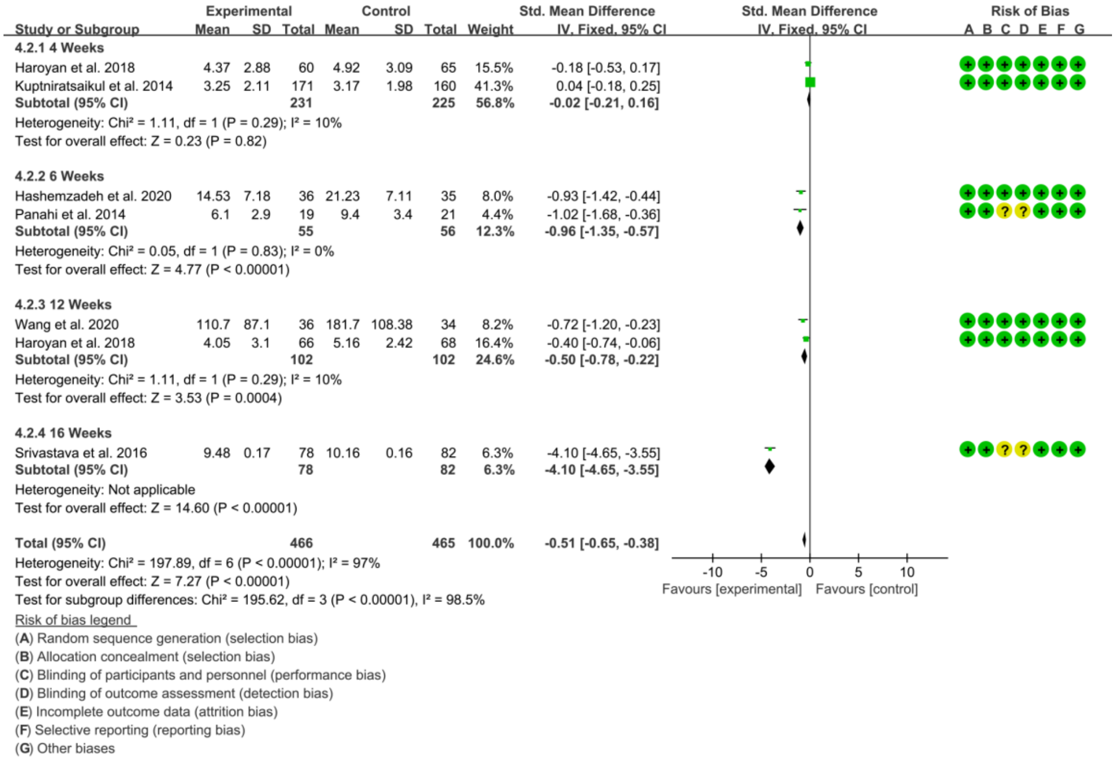

Risk of bias legend

(**A**) Random sequence generation (selection bias)
(**B**) Allocation concealment (selection bias)
(**C**) Blinding of participants and personnel (performance bias)
(**D**) Blinding of outcome assessment (detection bias)
(**E**) Incomplete outcome data (attrition bias)
(**F**) Selective reporting (reporting bias)
(**G**) Other biases

Study or Subgroup	Experimental Mean	Experimental SD	Experimental Total	Control Mean	Control SD	Control Total	Weight	Std. Mean Difference IV, Random, 95% CI	A	B	C	D	E	F	G
**4.3.4 4 Weeks**															
Kuptniratsaikul et al. 2014	3.41	2.09	171	3.26	2.05	160	17.2%	0.07 [-0.14, 0.29]	+	+	+	+	+	+	+
**Subtotal (95% CI)**			**171**			**160**	**17.2%**	**0.07 [-0.14, 0.29]**							
Heterogeneity: Not applicable															
Test for overall effect: Z = 0.66 (P = 0.51)															
**4.3.5 6 Weeks**															
Hashemzadeh et al. 2020	42.61	16.7	36	69.51	27.56	35	16.6%	-1.17 [-1.68, -0.67]	+	+	+	+	+	+	+
Panahi et al. 2014	18.7	10.3	19	30.4	9.4	21	16.1%	-1.17 [-1.84, -0.49]	+	+	?	?	+	+	+
**Subtotal (95% CI)**			**55**			**56**	**32.7%**	**-1.17 [-1.57, -0.76]**							
Heterogeneity: $Tau^2 = 0.00$; $Chi^2 = 0.00$, df = 1 (P = 0.99); $I^2 = 0\%$															
Test for overall effect: Z = 5.66 (P < 0.00001)															
**4.3.6 12 Weeks**															
Wang et al. 2020	398.4	401.61	36	676.6	325.16	34	16.6%	-0.75 [-1.24, -0.26]	+	+	+	+	+	+	+
Haroyan et al. 2018	4.37	9.02	66	7.16	12.76	68	17.0%	-0.25 [-0.59, 0.09]	+	+	+	+	+	+	+
**Subtotal (95% CI)**			**102**			**102**	**33.6%**	**-0.47 [-0.96, 0.02]**							
Heterogeneity: $Tau^2 = 0.08$; $Chi^2 = 2.73$, df = 1 (P = 0.10); $I^2 = 63\%$															
Test for overall effect: Z = 1.89 (P = 0.06)															
**4.3.7 16 Weeks**															
Srivastava et al. 2016	32.14	0.4	78	33.88	0.5	82	16.5%	-3.81 [-4.34, -3.29]	+	+	?	?	+	+	+
**Subtotal (95% CI)**			**78**			**82**	**16.5%**	**-3.81 [-4.34, -3.29]**							
Heterogeneity: Not applicable															
Test for overall effect: Z = 14.25 (P < 0.00001)															
**Total (95% CI)**			**406**			**400**	**100.0%**	**-1.17 [-2.20, -0.14]**							
Heterogeneity: $Tau^2 = 1.59$; $Chi^2 = 193.34$, df = 5 (P < 0.00001); $I^2 = 97\%$															
Test for overall effect: Z = 2.23 (P = 0.03)															
Test for subgroup differences: $Chi^2 = 189.44$, df = 3 (P < 0.00001), $I^2 = 98.4\%$															

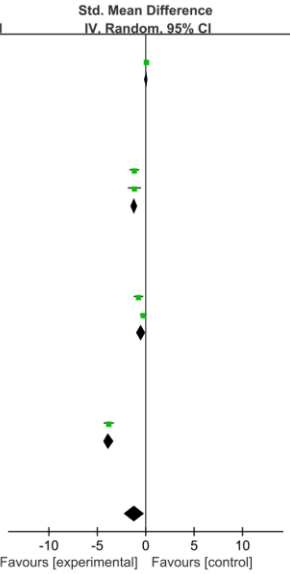

Risk of bias legend

(**A**) Random sequence generation (selection bias)
(**B**) Allocation concealment (selection bias)
(**C**) Blinding of participants and personnel (performance bias)
(**D**) Blinding of outcome assessment (detection bias)
(**E**) Incomplete outcome data (attrition bias)
(**F**) Selective reporting (reporting bias)
(**G**) Other biases

Study or Subgroup	Experimental Mean	Experimental SD	Experimental Total	Control Mean	Control SD	Control Total	Weight	Std. Mean Difference IV, Fixed, 95% CI	A	B	C	D	E	F	G
**4.4.4 4 Weeks**															
Kuptniratsaikul et al. 2014	3.28	2.38	171	3.16	2.36	160	41.6%	0.05 [-0.17, 0.27]	+	+	+	+	+	+	+
**Subtotal (95% CI)**			**171**			**160**	**41.6%**	**0.05 [-0.17, 0.27]**							
Heterogeneity: Not applicable															
Test for overall effect: Z = 0.46 (P = 0.65)															
**4.4.5 6 Weeks**															
Hashemzadeh et al. 2020	2.64	1.93	36	2.94	2.04	35	8.9%	-0.15 [-0.62, 0.32]	+	+	+	+	+	+	+
Panahi et al. 2014	0.15	0.5	19	0.76	0.9	21	4.6%	-0.81 [-1.46, -0.16]	+	+	?	?	+	+	+
**Subtotal (95% CI)**			**55**			**56**	**13.5%**	**-0.37 [-0.75, 0.00]**							
Heterogeneity: Chi² = 2.63, df = 1 (P = 0.10); I² = 62%															
Test for overall effect: Z = 1.94 (P = 0.05)															
**4.4.6 12 Weeks**															
Wang et al. 2020	48.6	42.14	36	76.2	53.64	34	8.4%	-0.57 [-1.05, -0.09]	+	+	+	+	+	+	+
Haroyan et al. 2018	1.58	1.7	66	1.95	1.76	68	16.8%	-0.21 [-0.55, 0.13]	+	+	+	+	+	+	+
**Subtotal (95% CI)**			**102**			**102**	**25.2%**	**-0.33 [-0.61, -0.05]**							
Heterogeneity: Chi² = 1.41, df = 1 (P = 0.24); I² = 29%															
Test for overall effect: Z = 2.35 (P = 0.02)															
**4.4.7 16 Weeks**															
Srivastava et al. 2016	4.08	0.17	78	4.16	0.18	82	19.6%	-0.45 [-0.77, -0.14]	+	+	?	?	+	+	+
**Subtotal (95% CI)**			**78**			**82**	**19.6%**	**-0.45 [-0.77, -0.14]**							
Heterogeneity: Not applicable															
Test for overall effect: Z = 2.84 (P = 0.005)															
**Total (95% CI)**			**406**			**400**	**100.0%**	**-0.20 [-0.34, -0.06]**							

Heterogeneity: Chi² = 13.43, df = 5 (P = 0.02); I² = 63%
Test for overall effect: Z = 2.85 (P = 0.004)
Test for subgroup differences: Chi² = 9.39, df = 3 (P = 0.02), I² = 68.1%

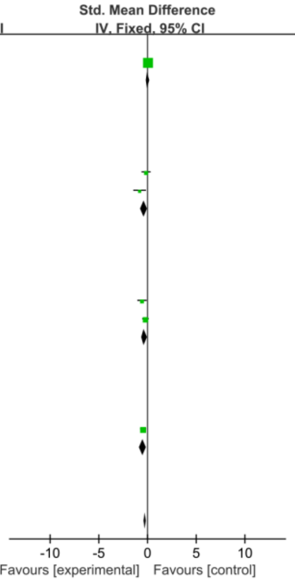

Risk of bias legend
(**A**) Random sequence generation (selection bias)
(**B**) Allocation concealment (selection bias)
(**C**) Blinding of participants and personnel (performance bias)
(**D**) Blinding of outcome assessment (detection bias)
(**E**) Incomplete outcome data (attrition bias)
(**F**) Selective reporting (reporting bias)
(**G**) Other biases

Study or Subgroup	Experimental Events	Experimental Total	Control Events	Control Total	Weight	Risk Ratio M-H, Random, 95% CI
**4.7.1 4 Weeks**						
Shep et al. 2019	9	70	26	69	11.9%	0.34 [0.17, 0.67]
Kuptniratsaikul et al. 2014	55	171	65	160	21.5%	0.79 [0.59, 1.06]
**Subtotal (95% CI)**		**241**		**229**	**33.4%**	**0.55 [0.24, 1.26]**
Total events	64		91			
Heterogeneity: Tau² = 0.29; Chi² = 5.10, df = 1 (P = 0.02); I² = 80%						
Test for overall effect: Z = 1.41 (P = 0.16)						
**4.7.2 6 Weeks**						
Jamali et al. 2020	0	36	0	36		Not estimable
Hashemzadeh et al. 2020	0	36	0	35		Not estimable
Kuptniratsaikul et al. 2009	16	45	33	46	17.6%	0.50 [0.32, 0.76]
Panahi et al. 2014	3	19	4	21	4.6%	0.83 [0.21, 3.24]
Madhu et al. 2013a	2	30	2	30	2.6%	1.00 [0.15, 6.64]
Madhu et al. 2013b	4	30	5	30	5.5%	0.80 [0.24, 2.69]
**Subtotal (95% CI)**		**196**		**198**	**30.2%**	**0.56 [0.38, 0.82]**
Total events	25		44			
Heterogeneity: Tau² = 0.00; Chi² = 1.37, df = 3 (P = 0.71); I² = 0%						
Test for overall effect: Z = 3.00 (P = 0.003)						
**4.7.3 8 Weeks**						
Nakagawa et al. 2014	0	18	0	23		Not estimable
**Subtotal (95% CI)**		**18**		**23**		**Not estimable**
Total events	0		0			
Heterogeneity: Not applicable						
Test for overall effect: Not applicable						
**4.7.4 12 Weeks**						
Henrotin et al. 2019a	18	49	3	23	6.2%	2.82 [0.92, 8.61]
Henrotin et al. 2019b	10	47	3	22	5.7%	1.56 [0.48, 5.11]
Haroyan et al. 2018	7	66	4	68	5.7%	1.80 [0.55, 5.87]
Wang et al. 2020	14	36	18	34	15.5%	0.73 [0.44, 1.23]
**Subtotal (95% CI)**		**198**		**147**	**33.1%**	**1.38 [0.68, 2.81]**
Total events	49		28			
Heterogeneity: Tau² = 0.28; Chi² = 6.64, df = 3 (P = 0.08); I² = 55%						
Test for overall effect: Z = 0.88 (P = 0.38)						
**4.7.5 16 Weeks**						
Srivastava et al. 2016	2	78	4	82	3.2%	0.53 [0.10, 2.79]
**Subtotal (95% CI)**		**78**		**82**	**3.2%**	**0.53 [0.10, 2.79]**
Total events	2		4			
Heterogeneity: Not applicable						
Test for overall effect: Z = 0.76 (P = 0.45)						
**Total (95% CI)**		**731**		**679**	**100.0%**	**0.77 [0.56, 1.05]**
Total events	140		167			
Heterogeneity: Tau² = 0.10; Chi² = 18.03, df = 10 (P = 0.05); I² = 45%						
Test for overall effect: Z = 1.64 (P = 0.10)						
Test for subgroup differences: Chi² = 5.11, df = 3 (P = 0.16), I² = 41.3%						

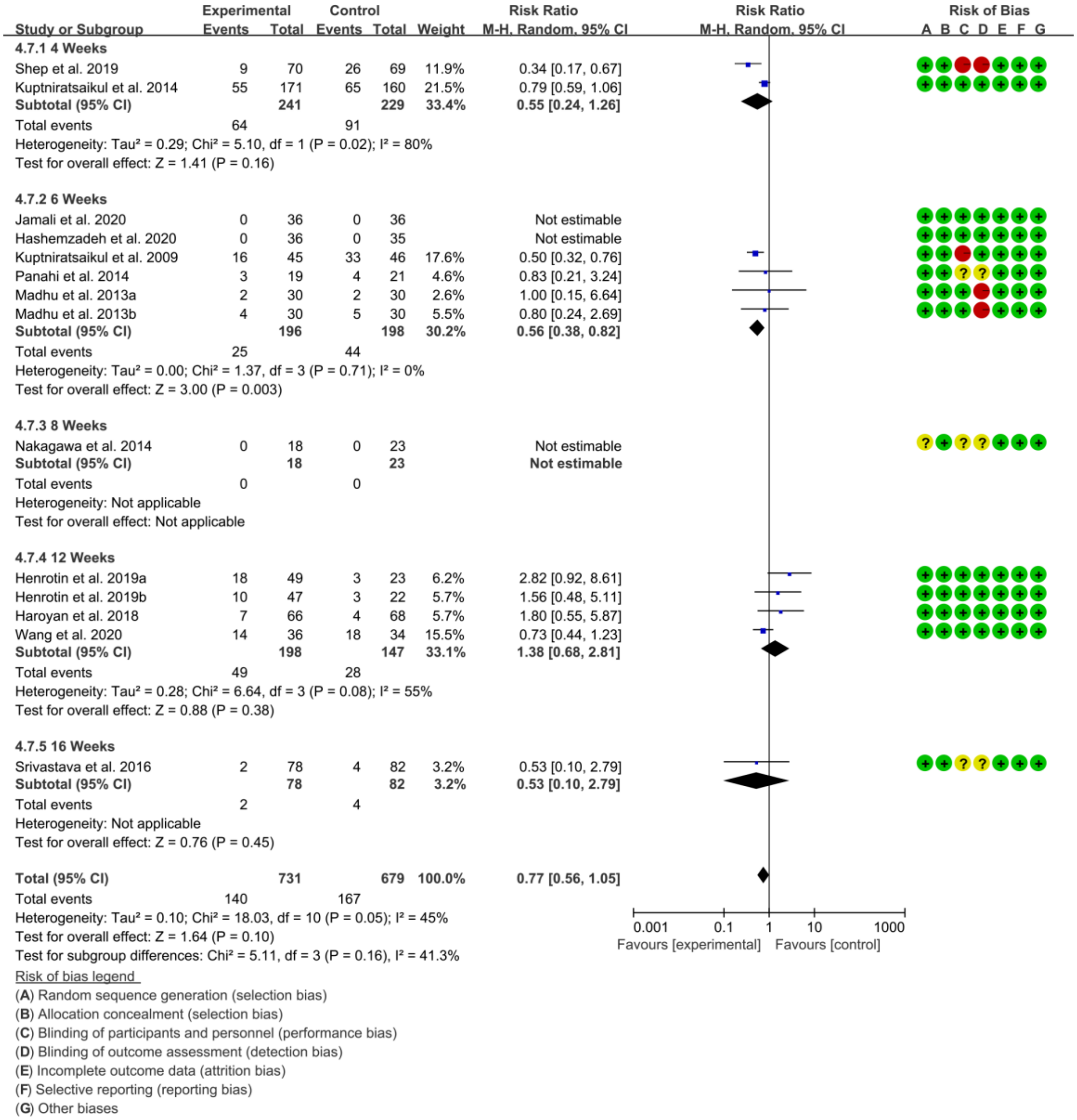

Risk of bias legend

(**A**) Random sequence generation (selection bias)
(**B**) Allocation concealment (selection bias)
(**C**) Blinding of participants and personnel (performance bias)
(**D**) Blinding of outcome assessment (detection bias)
(**E**) Incomplete outcome data (attrition bias)
(**F**) Selective reporting (reporting bias)
(**G**) Other biases

Table S1. Search Strategies for Pubmed and Embase

**PubMed**	(Curcumin OR Curcumas OR Tumeric OR Tumerics OR Turmeric OR Turmerics OR Curcuma zedoaria OR Curcuma zedoarias OR zedoaria, Curcuma OR Zedoary zedoaria OR Zedoary zedoarias OR zedoaria, Zedoary OR Curcuma longa OR Curcuma longas OR longa, Curcuma OR Curcuma Longa)<br>AND<br>(Osteoarthritis OR Osteoarthritides OR Osteoarthrosis OR Osteoarthroses OR Arthritis, Degenerative OR Arthritides, Degenerative OR Degenerative Arthritides OR Degenerative Arthritis OR Osteoarthrosis Deformans)<br>AND<br>(random* controlled trial [pt] OR controlled clinical trial* [pt] OR randomized [tiab] OR placebo [tiab] OR drug therapy [sh] OR random* [tiab] OR trial* [tiab] OR group* [tiab])<br>NOT<br>(animals [mh] NOT humans [mh])
**EMBASE**	1 'Curcumin' OR 'Curcumas'<br>2 'Tumeric' OR 'Tumerics' OR 'Turmeric' OR 'Turmerics'<br>3 'Zedoary zedoaria' OR 'Zedoary zedoarias Curcuma longa'<br>4 'Curcuma zedoaria' OR 'Curcuma zedoarias' OR 'Curcuma longas' OR 'Curcuma Longa'<br>5 1 or 2 or 3 or 4<br>6 'Osteoarthritis'/exp<br>7 'Osteoarthritides' or 'Osteoarthrosis' or 'Osteoarthroses'<br>8 'Degenerative Arthritides' OR 'Degenerative Arthritis' OR 'Osteoarthrosis Deformans'<br>9 6 or 7 or 8<br>10 5 and 9<br>11 'randomized controlled trial'<br>12 'single blind procedure' or 'double blind procedure'<br>13 'crossover procedure'<br>14 12 or 13 or 14<br>15 10 and 14
